# Investigation of NF-****κ****B1 and NF-****κ****BIA Gene Polymorphism in Non-Small Cell Lung Cancer

**DOI:** 10.1155/2014/530381

**Published:** 2014-02-23

**Authors:** Y. M. Oltulu, E. Coskunpinar, G. Ozkan, E. Aynaci, P. Yildiz, T. Isbir, I. Yaylim

**Affiliations:** ^1^Department of Molecular Medicine, Institute of Experimental Medicine Research, Istanbul University DETAE, P.O. Box 7, 34390 Çapa/İstanbul, Turkey; ^2^Department of Chest Diseases, Faculty of Medicine, Medipol University, Istanbul, Turkey; ^3^Third Clinics, Yedikule Chest Diseases and Thoracic Surgery Training Research Hospital, Istanbul, Turkey; ^4^Department of Medical Biology, School of Medicine, Yeditepe University, Istanbul, Turkey

## Abstract

Lung cancer is a complex, multifactorial disease which is the leading cause of cancer death in both men and women. NF-**κ**B is a transcription factor which is known to affect the expression of more than 150 genes related to inflammation, lymphocyte activation, cell proliferation, differentiation, and apoptosis, as well as contributing to cell apoptosis and survival. However, NF-**κ**BIA (I**κ**B**α**) is the inhibitor of the transcription factor. The -94ins/delATTG polymorphism of the NF-**κ**B1 gene promoter region which causes a functional effect and NF-**κ**BIA 3′UTR A → G polymorphism has been shown to be related to various inflammatory diseases and cancer. Ninety-five NSCLC patients and 99 healthy controls were included in study. The NF-**κ**B1 -94ins/delATTG and NF-**κ**BIA 3′UTR A → G polymorphism have been studied by using PCR-RFLP method. It was found that the NF-**κ**B1 -94ins/delATTG DD genotype and D allele frequencies were higher in patients than healthy controls and the presence of the DD genotype has a 3.5-fold increased risk of the disease (*P*: 0.014). This study is the first to investigate the NF-**κ**B1 -94ins/delATTG and NF-**κ**BIA 3′UTR A → G polymorphism together in the Turkish population. According to the results, the NF-**κ**B1 -94ins/del ATTG promoter polymorphism may have a role in lung carcinogenesis and prognosis.

## 1. Introduction

Non-small cell lung cancer (NSCLC), which includes squamous cell carcinoma, adenocarcinoma, and large-cell carcinoma, is the most common lung cancer possessing approximately 80–85% rates in the prevalence of lung cancer case [1]. Despite all advances in the current treatments including surgical resection, chemotherapy, and radiation therapy alone or in combination, the disease is rarely curable prognosis and remains poor [2]. As all of these facts are considered, recent researches are tending to understand molecular, biological, and genetic factors and to find other prognostic factors providing long-term survival and intending new targeted therapies [3, 4]. Lung cancer cells manage to escape from the signal transduction pathways to facilitate their own survival and proliferation by using multiple mechanisms [5]. Carcinogens and inflammatory cytokines contributing substantially to cancer development are involved in activation of common cell survival signaling pathways. The one of this cell survival signal is nuclear factor-kappaB (NF-*κ*B) which is involved in multiple steps in carcinogenesis and in cancer cell's resistance to chemo- and radiotherapy [6]. Recently, many studies with animal models and cell culture systems indicate the interplay between NF-*κ*B and lung carcinogenesis, which emphasizes the importance of targeting the NF-*κ*B signaling pathway for lung cancer treatment and chemoprevention [7]. NF-*κ*B, a nuclear transcription factor [8], was first identified in 1986 by Sen and Baltimore [9]. It was initially observed to be a transcription factor binding to the intronic enhancer of the kappa light chain gene (the *κ*B site) in B cells, but it was later shown to be present in every cell type [10]. Afterwards, NF-*κ*B emerged as a major regulator of more than 200 genes involved in diverse process such as cell survival and cell adhesion, inflammation, differentiation, and growth [11]. NF-*κ*B is activated by phosphorylation of I*κ*B*α* which is catalyzed by an I*κ*B*α* Kinase (IKK) complex consisting of IkK-*α*, IkK-*β*, IkK-*γ* (also called NEMO), and other proteins yet to be identified [12, 13]. After activation of NF-*κ*B, I*κ*B*α* is degraded and p50–p65 heterodimer is translocated to the nucleus, binds to the DNA (at the promoter region), and activates gene [14, 15]. Currently, studies show that activation of the transcription factor nuclear factor (NF) *κ*B is a novel mechanism of chemoresistance in NSCLC and other tumors [16, 17]. NF-*κ*B1 is inhibited by IkB proteins (e.g., NF-*κ*BIA) [18]. Phosphorylation of serine residues on the I-kappa-B proteins, by kinases and marks them for degradation, thereby allowing activation of the NF-*κ*B complex [19]. As the first potential functional NF-*κ*B1 genetic variation was identified 94 insertion/deletion ATTG located between two received key promoter regulatory elements in the NF-*κ*B1 gene, ATTG deletion causes the loss of binding to nuclear proteins, which leads to reduced promoter activity [20]. The NF-*κ*B -94ins/delATTG polymorphism of the NF-*κ*B1 gene promoter region which causes a functional effect and NF-*κ*BIA 3′UTR A → G polymorphism have been shown to be associated with various inflammatory diseases and cancers [21]. The aim of this study is firstly to investigate the NF-*κ*B1 -94ins/delATTG and NF-*κ*BIA 3′UTR A → G polymorphism together in the Turkish population.

## 2. Materials and Methods

### 2.1. Study Groups

Ninety-five primary non-small cell lung cancer (NSCLC) patients and 99 healthy individuals were included in the study. NSCLC patients were recruited from the Yedikule Chest Diseases and Thoracic Surgery Training Research Hospital, Istanbul. The diagnosis of NSCLC was made by the pathologist based on histopathological examination. In NSCLC group, all subjects were diagnosed and confirmed with histopathological examination. They were all newly diagnosed without a history of prior radiotherapy and/or chemotherapy. Exclusion criteria included primary extra pulmonary malignancy, small cell lung cancer, a history of malignant disease, and withdrawal of consent and patient aged less than 18. Pathological staging information on all NSCLC cases was confirmed by manual review of the pathology reports and clinical charts. Nodal status was categorized as no regional lymph nodes affected (N0) or at least one nodal metastasis. The mean ages of the patients and controls were 61.2 ± 9.83 years and 57.49 ± 10.83 years, respectively. The percentage of females was 6.3% for patients and 32.3% for controls, and percentage of males was 93.7% for patients and 67.7% for controls. 99 healthy subjects without any malignancy were selected for the control group that is comprised only of individuals with a negative family history of cancer. The patient and control groups were matched for age. All participants signed an informed consent before enrollment and Institutional Ethical committee approval was obtained for the study.

### 2.2. Polymorphism Analysis

**Figure 1:**
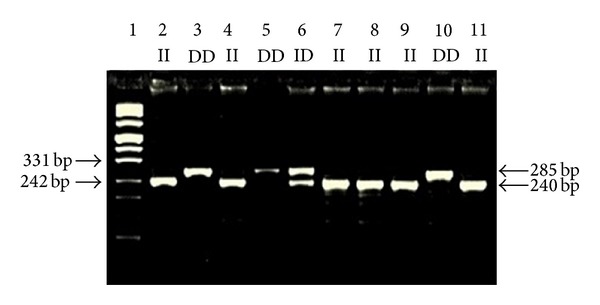
Representative genotypes of NF-*κ*B1 -94ins/delATTG polymorphism. Lane 1: marker DNA ladder; Lanes 2, 4, 7, 8, 9, and 11: ins/ins (ATTG2/ATTG2) genotypes; Lanes 3, 5, and 10: del/del (ATTG1/ATTG1) genotypes; Lane 6: heterozygous del/ins (ATTG1/ATTG2) genotypes.

**Figure 2:**
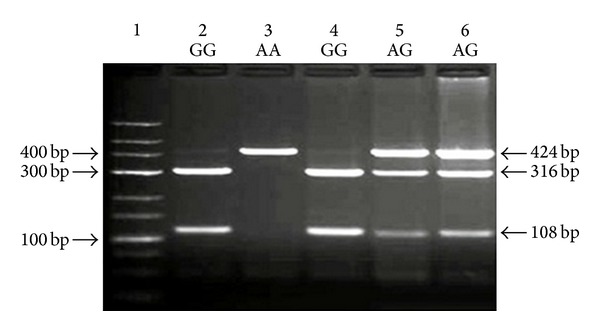
Representative genotypes of NF-*κ*BIA 3′UTR A → G polymorphism. Lane 1: marker DNA ladder; Lanes 2 and 4: GG mutant genotypes; Lane 3: AA wild-type homozygous genotypes; Lanes 5 and 6: AG heterozygous genotypes.

Blood samples from all study participants were collected in EDTA-containing tubes. Genomic DNA was extracted from peripheral whole blood according to kit protocol (High Pure PCR Template Preparation Kit, REF 11796828001 (Roche, Diagnostics GmbH, Mannheim, Germany). Genotyping was performed by polymerase chain reaction (PCR) and restriction fragment length polymorphism (RFLP); the procedures of PCR-RFLP are given in Table 1. Two separate PCR reactions were used to detect the two types of polymorphism in NF-*κ*B gene, namely, NF-*κ*B1 -94ins/delATTG polymorphism and NF-*κ*BIA 3′UTR A → G polymorphism. The appropriate primers were used to amplify the corresponding gene of the subjects by PCR and the reaction products were digested by using the appropriate enzyme and incubated at 37°C overnight. The digested products were analyzed on 3% agarose gel, stained with ethidium bromide, and examined under transillumination (Figures 1 and 2). Each gel was read by two observers, unaware of the subject's status. In order to verify our PCR-RFLP results, we repeated PCR-RFLP stage 2 times for each of selected subject. The expected results after restriction for each gene were also given in Table 1.

### 2.3. Statistical Analysis

Statistical analyses were performed using the SPSS software package (revision 13.0 SPSS Inc., Chicago, IL, USA). Data were expressed as means ± SD. Differences in the distribution of NF-*κ*B1 -94ins/delATTG and NF-*κ*BIA 3′UTR A → G genotypes or alleles between cases and controls were tested using the Chi-square statistic, respectively (Table 2). Differences in characteristics between NSCLC patients and controls were assessed with Fisher's exact test, as well as disparities in genotype and allele frequencies. Relative risk at 95% confidence intervals (CI) was calculated as the odds ratio (OR). Values *P* < 0.05 were considered statistically significant. A multivariate logistic regression model was performed to investigate possible effects of genotypes and alleles after adjustment for age.

## 3. Results

In this study, we examined 194 volunteers, 95 NSCLC (89 males; 6 females) patients, and 99 healthy people (67 males; 32 females) detecting any chronic disease or any evidence of malignancy. Distribution of NF-*κ*B1 and NF-*κ*BIA genotypes according to clinic features in NSCLC patients is shown in Table 3. The distribution of the NF-*κ*B1 -94ins/delATTG genotypes in control and NSCLC patients was found to be significantly different (*P*: 0.048). It was evaluated that individuals carrying DD genotype had 3.5-fold increased risk for NSCLC (*P*: 0.014 *χ* ^2^: 5.605, O.R: 3.50, %95 CI: 1.24–9.87). No statistically significant differences between groups were observed when the NF-*κ*BIA 3′UTR A → G genotypes distributions were compared (*P*: 0.844). A significant correlation between genotype combinations of NF-*κ*B1 and NF-*κ*BIA (DDAG genotype) and NSCLC risk was found compared to all other combinations (*P*: 0.025; O.R: 5.035; 95% CI: 1.067–24.14) and DDAG genotype had increased risk for NSCLC. The prevalence of IIAA genotype combinations versus to all other combinations was 5.3% in patients and 12.1% in the control group, but there are no statistically significant differences between groups (*P*: 0.091; O.R: 0.403; 95% CI: 0.136–1.191). The results of multivariate logistic regression analysis are presented in Table 4. Gender, age (<57/≥57), and NF-*κ*B1 DD genotype were associated with NSCLC in univariate analysis, and additionally these were associated with this disease in multivariate logistic regression analysis.

## 4. Discussion

A functional polymorphism in the NF-*κ*B1 gene promoter region (-94ins/delATTG) has been identified and associated with both chronic inflammatory diseases and malignant diseases [22]. NF-*κ*B is inactivated in the cytoplasm by I*κ*B*α*, *β*, or *γ* and the most common protein of this family is the NF-*κ*B inhibitor *α* (NF-*κ*BIA) [23]. -94ins/delATTG polymorphism has evidence from two independent functional assays, in vitro promoter activity and differential an unidentified nuclear protein binding, that the specific allele inherited likely has functional consequences [24]. NF-*κ*BIA 3′UTR A → G polymorphism may affect mRNA stability and translational efficacy or conduces to differential nuclear RNA processing, or export also cannot be completely excluded. Many studies have been conducted to investigate a possible association between NF-*κ*B1 -94ins/delATTG and NF-*κ*BIA 3′UTR A → G polymorphism and both inflammatory diseases and various cancer types [25]. However no data are available in the English literature to report the association with NSCLC to date. Our study is the initial report on these two forms of polymorphism (both NF-*κ*B1 -94ins/delATTG and NF-*κ*BIA 3′UTR A → G) studied together in NSCLC patients to our knowledge. The genotypic combinations of NF-*κ*B1 and NF-*κ*B2 polymorphism have been shown to be associated with the development of common inflammatory diseases including ulcerative colitis (UC), Crohn's disease, and Type I diabetes, as well as susceptibility of several cancers, such as oral squamous cell carcinoma and colorectal cancer [26]. It can be concluded that previous studies have conflicting results [27]. Oliver et al. suggest that the NF-*κ*B1 -94ins/delATTG gene variation, previously associated with UC susceptibility in North Americans, does not influence either susceptibility or phenotype of UC in the Spanish population [28]. In this study, we performed a risk association between the NF-*κ*B1 -94ins/delATTG promoter polymorphism and NSCLC. The -94ins/delATTG polymorphism has been shown as a first potential functional NF-*κ*B1 polymorphism by Karban et al. Nuclear proteins from normal human colon tissue showed significant binding to -94insATTG but not to -94delATTG containing oligonucleotides. NF-*κ*B1 promoter/exon 1 luciferase reporter plasmid constructs containing the -94delATTG allele and transfected into either HeLa or HT-29 cell lines showed low promoter activity more than comparable constructs containing the -94insATTG allele. Therefore, it is known that D allele promoter activity is low and I allele promoter activity is high. Previous studies have suggested that D allele may result in decreased NF-*κ*B1 message and hence decreased p50/p105 NF-*κ*B protein production leads to increased inflammatory response. Otherwise, a potential explanation of decreased NF-*κ*B1 D allele gene expression may be the resulting decreases in NF-*κ*B p50/p65 heterodimers that are major mediators of inflammation [29].

Defects in components that regulate NF-*κ*B release from I*κ*B*α* result in constitutive or decreased NF-*κ*B activation. These components may be any of the kinases, phosphatases, or other signal transducers, normally involved in NF-*κ*B-activation pathways [30]. Sonenshein suggests that alterations of NF-*κ*B1 expression play an important role in the protection of cells from apoptosis [31]. NF-*κ*B1 activity has been observed in various types of cancer, as well as colorectal cancer and breast cancer, to contribute to tumor angiogenesis, invasion, and progression [32]. Therefore, the variants of the NF-*κ*B1 gene could be expected to have an effect on cell death and thus carcinogenesis. NF-*κ*B1 3′UTR A → G polymorphism has functional effects on expression of the NF-*κ*BIA gene and altered NF-*κ*B transcription [33]. There are many studies with different results on NF-*κ*BIA 3′UTR polymorphism in the literature [34]. Our results suggested that NF-*κ*BIA polymorphism has no effect on risk of NSCLC. In conclusion, we here clearly demonstrated that NF-*κ*B1 -94ins/delATTG promoter polymorphism and the presence of the DD genotype might have a risk factor for NSCLC pathogenesis in our ethnic population. Larger trials that included different ethnic groups are necessary to define objectively the correlation between NF-*κ*B1 -94ins/delATTG promoter and development of NSCLC as well as prognosis of disease.

## Figures and Tables

**Table 1:** PCR and RFLP procedures and products of NF-*κ*B1 -94ins/delATTG and NF-*κ*BIA 3′UTR A→G.

	Primers (forward and reverse)	PCR product	Restriction enzyme	Restriction products
NF-*κ*B1	5′-TGGGCACAAGTCGTTTATGA-3′	285 bp	Van91l (PflMI)	ATTG_2_/ATTG_2_ (ins/ins): 240 bp, 45 bp
	5′-CTGGAGCCGGTAGGGAAG-3′			ATTG_1_/ATTG_1_ (del/del): 281
				ATTG_2_/ATTG_1_ (ins/del): 281, 240, 45
NF-*κ*BIA	5′-GGCTGAAAGAACATGGACTTG-3′	424 bp	HaeIII	AA wild type: 424
	5′-GTACACCATTTACAGGAGGG-3′			AG heterozygous: 316, 108
				GG mutant: 424, 316, 108

**Table 2:** Distribution of NF-*κ*B1 and NF-*κ*BIA genotypes and allele in NSCLC patients and controls.

Genotypes/alleles	Controls *n* (%)	Patients *n* (%)	O.R (95% CI)	*P* value
NF-*κ*B1				
II	46 (46.47)	35 (36.84)	Reference	
ID	47 (47.47)	44 (46.32)	1.23 (0.67–2.25)	0.500
DD	6 (6.06)	16 (16.84)	3.50 (1.24–9.87)	0.014
ID + DD	53 (53.53)	60 (63.16)	1.49 (0.84–2.64)	0.174
I allele	139 (70.2)	114 (60)	Reference	
D allele	59 (29.8)	76 (40)	1.57 (1.03–2.39)	0.035
NF-*κ*BIA				
AA	21 (21.21)	17 (17.9)	Reference	
AG	45 (45.46)	45 (47.36)	1.24 (0.58–2.65)	0.587
GG	33 (33.33)	33 (34.74)	1.24 (0.55–2.75)	0.605
AG + GG	78 (78.79)	78 (82.1)	1.24 (0.61–2.52)	0.561
A allele	87 (43.94)	79 (41.58)	Reference	
G allele	111 (56.06)	111 (58.42)	1.10 (0.74–1.65)	0.639

O.R: Odds ratio; CI: confidence interval.

**Table 3:** Distribution of NF-*κ*B1 and NF-*κ*BIA genotypes with clinic features in NSCLC patients.

	NF-*κ*B1				NF-*κ*BIA			
	II	ID	DD	*P* value	AA	AG	GG	*P* value
	*n* (%)	*n* (%)	*n* (%)		*n* (%)	*n* (%)	*n* (%)	
Sex								
Men	33 (37.10)	41 (46.10)	15 (16.80)	0.980	15 (16.90)	43 (48.30)	31 (34.80)	0.570
Women	2 (33.30)	3 (50.00)	1 (16.70)		2 (33.30)	2 (33.30)	2 (33.30)	
Age								
<57	10 (29.4)	17 (50)	7 (20.6)	0.496	5 (14.7)	19 (55.9)	10 (29.4)	0.567
≥57	27 (41.5)	27 (41.5)	11 (16.9)		12 (18.5)	29 (44.6)	24 (36.9)	
Smoking (box/year)								
<50	25 (48.10)	24 (46.20)	3 (5.80)	0.02	10 (19.20)	25 (48.10)	17 (32.70)	0.876
≥50	10 (23.30)	20 (46.50)	13 (30.20)		7 (16.30)	20 (46.50)	16 (37.20)	
Alcohol consumption								
No	17 (31.50)	28 (51.90)	9 (16.70)	0.405	6 (11.10)	31 (57.40)	17 (31.50)	0.044
Yes	18 (43.90)	16 (39.00)	7 (17.10)		11 (26.80)	14 (34.10)	16 (39.00)	
Histopathology								
Squamous	16 (45.70)	16 (45.70)	3 (8.60)	0.179	7 (20.00)	20 (57.10)	8 (22.90)	0.173
Nonsquamous	19 (31.70)	28 (46.70)	13 (21.70)		10 (16.70)	25 (41.70)	25 (41.70)	
Total protein								
<6 g/L	2 (50.00)	0 (0)	2 (50.00)	0.087	0 (0)	3 (75.00)	1 (25.00)	0.452
≥6 g/L	31 (38.30)	38 (46.90)	12 (14.80)		16 (19.80)	37 (45.70)	28 (34.60)	
Albumin								
<3 g/L	6 (54.50)	1 (9.10)	4 (36.40)	0.019	1 (9.10)	6 (54.50)	4 (36.40)	0.635
≥3 g/L	26 (34.70)	39 (52.00)	10 (13.30)		16 (21.30)	35 (46.70)	24 (32.00)	
Calcium								
<10 mg/Dl	30 (40.00)	30 (40.00)	15 (20.00)	0.001	16 (21.30)	35 (46.70)	24 (32.00)	0.506
≥10 mg/dL	0 (0)	11 (100)	0 (0)		1 (9.10)	7 (63.60)	3 (27.30)	
LDH								
<250 U/L	22 (36.10)	28 (45.90)	11 (18.00)	0.644	13 (21.30)	31 (50.80)	17 (27.90)	0.249
≥250 U/L	10 (47.60)	8 (38.10)	3 (14.30)		3 (14.30)	8 (38.10)	10 (47.60)	

**Table 4:** The results of multivariate logistic regression.

Covariates	*P* value	Exp (B)	95% C.I for Exp (B)
Gender	<0.001	7.866	2.915–21.231
Age (<57/≥57)	<0.001	5.074	2.633–9.776
NF-*κ*B1 DD genotype	0.035	3.167	1.086–9.234
